# Corrosion Behavior of Al Modified with Zn in Chloride Solution

**DOI:** 10.3390/ma15124229

**Published:** 2022-06-15

**Authors:** Jesús Porcayo Calderón, José Luis Reyes Barragán, Jesús Israel Barraza Fierro, Héctor Cruz Mejía, Cinthya Dinorah Arrieta González, Víctor Ravelero Vázquez, Kevin Piedad Sánchez, María Teresa Torres-Mancera, Rogel Fernando Retes-Mantilla, Roberto Ademar Rodríguez-Díaz

**Affiliations:** 1 Departamento de Ingeniería Química y Metalurgia, Universidad de Sonora, Hermosillo 83000, Mexico; jporcayoc@gmail.com; 2 Departamento de Ingeniería en Diseño, Universidad Politécnica de la Zona Metropolitana de Guadalajara, Tlajomulco de Zúñiga 45640, Mexico; jlbecario@yahoo.com (J.L.R.B.); victor.ravelero@gmail.com (V.R.V.); 3 Escuela de Preparatoria, Universidad La Salle Nezahualcóyotl, Nezahualcóyotl 57300, Mexico; jbarraza@ulsaneza.edu.mx; 4 Departamento de Ciencias Básicas, TecNM Campus Tláhuac II, Camino Real 625, Jardines de Llano, Tláhuac, San Juan Ixtayopan 13550, Mexico; 5 División de Ingeniería en Nanotecnología, Universidad Politécnica del Valle de México, Av. Mexiquense s/n, esq., Av. Universidad Politécnica, Villa Esmeralda, Fuentes del Valle 54910, Mexico; hector.cruz@upvm.edu.mx; 6 Facultad de Química, Universidad Nacional Autónoma de México, Edificio D, Circuito de los Institutos s/n, Cd. Universitaria, Ciudad de México 04510, Mexico; 7 Tecnológico Nacional de México—Instituto Tecnológico de Zacatepec, Calzada Instituto Tecnológico 27, Zacatepec 62780, Mexico; cdaglez@gmail.com; 8 Departamento de Ingeniería Química, Tecnológico de Estudios Superiores de Coacalco, Av. 16 de Septiembre 54, Coacalco 55700, Mexico; kevinpiedad1@gmail.com; 9 Departamento de Ingeniería Química y Ambiental, Tecnológico de Estudios Superiores de Coacalco, Av. 16 de Septiembre 54, Coacalco 55700, Mexico; teresa@tesco.edu.mx; 10 Departamento de Posgrado en Logística y Cadena de Suministro, Tecnológico de Estudios Superiores de Coacalco, Av. 16 de Septiembre 54, Coacalco 55700, Mexico; retes@tesco.edu.mx; 11 Departamento de Ingeniería de Materiales, Tecnológico de Estudios Superiores de Coacalco, Av. 16 de Septiembre 54, Coacalco 55700, Mexico

**Keywords:** Al alloy, Al-Zn alloy, corrosion, electrochemical techniques

## Abstract

Aluminum-based alloys have been considered candidate materials for cathodic protection anodes. However, the Al-based alloys can form a layer of alumina, which is a drawback in a sacrificial anode. The anodes must exhibit uniform corrosion to achieve better performance. Aluminum can be alloyed with Zn to improve their performance. In this sense, in the present research, the electrochemical corrosion performance of Al-xZn alloys (x = 1.5, 3.5, and 5 at.% Zn) exposed to 3.5 wt.% NaCl for 24 h was evaluated. Polarization curves, linear polarization resistance (LPR), and electrochemical impedance spectroscopy (EIS) were used to identify the electrochemical behavior. The microstructure of the samples before the corrosion assessment was characterized by means of X-ray diffraction analyses (XRD) and scanning electron microscopy (SEM). In addition, microstructures of the corroded surfaces were characterized using X-ray mappings via SEM. Polarization curves indicated that Zn additions changed the pseudo-passivation behavior from what pure Al exhibited in a uniform dissolution regime. Furthermore, the addition of Zn shifted the corrosion potential to the active side and increased the corrosion rate. This behavior was consistent with the proportional decrease in polarization resistance (R_p_) and charge transfer resistance (*R_ct_*) in the EIS. The analysis of EIS was done using a mathematical model related to an adsorption electrochemical mechanism. The adsorption of chloride at the Al-Zn alloy surface formed aluminum chloride intermediates, which controlled the rate of the process. The rate constants of the reactions of a proposed chemical mechanism were evaluated.

## 1. Introduction

Aluminum alloys are widely applied in spacecraft and automotive industries due to their high strength and low density. In addition, Al materials have good corrosion resistance in several environments, which originates from the aluminum oxide that develops on the substrate. The alumina film induces a passivation effect on the metallic substrate. However, if the corrosion potential overcomes the pitting potential, localized corrosion can activate the aluminum surface. The Al substrate can be corroded due to aggressive anions, such as chloride in seawater [1].

Al alloys immersed in chloride environments can be prone to pitting corrosion and this behavior could be enhanced by the precipitation of secondary-phase particles. Pitting corrosion is one of the main mechanisms for the damage and failure of high-strength Al alloys [2]. Pitting corrosion has a mechanism with four stages: processes that occur at the products–electrolyte interface, vacancy movement inside the passive film, generation of metastable pits, and stable pit growth. These stages depend on the chemical composition and structure of the oxide film [3].

Al-based materials have been considered for cathodic protection as sacrificial anodes [4]. The sacrificial anodes must have a high oxidation current density, homogeneous degradation on the surface, and slight or a lack of protective film formation. Aluminum samples accomplished two of these features, but their ability to form alumina is a drawback. Additionally, the pitting process is another disadvantage. In this regard, low alloying with other elements, such as Zn, Mg, Sn, and Cu [5,6], is used to shift the potential toward the electronegative side and generate a more uniform attack [4].

It is worth noticing that cathodic protection (CP) is an electrochemical technique that is used to control the corrosion of iron and steels. This technological advance is applied at the international level and in various industries to protect metallic structures, such as ship hulls, buried piping, heat exchangers, and marine structures. The cathodic protection is essential for polarizing metal structures, which necessitate corrosion protection in the cathodic direction by providing an electrical current; alternatively, it can be attained by putting a galvanic component in contact with a sacrificial anode. 

Zn and Mg are more active than Al; accordingly, these additions promote a decrease in the corrosion potential. In addition, Zn or Sn shift the pitting potential of Al to more negative values, which allows for using them as anodes in cathodic protection systems [7]. In this respect, it is essential to distinguish whether these effects are given by precipitated compounds in interdendritic areas or grain boundary zones, or by the solid solution of these elements in an Al matrix. Generally, Al sacrificial anodes are based on the binary alloy Al-Zn system [8,9]. However, Al-Zn electrodes have not been studied extensively.

In Al-Zn alloys, Zn is usually precipitated at grain boundaries or interdendritic regions [9]. Polarization tests on these alloys promote galvanic and pitting due to the local composition variation, which, in turn, induces a diminution of the anode efficiency [3]. Valdés et al. [10] developed three Al-based alloys with Zn (4.1–7.77 at.%), Mg (5.84–10.67 at.%), and Li (0.12–0.15 at.%). They revealed a dendritic matrix of an Al(α) solid solution, with Al_2_Mg_3_Zn_3_, Mg_7_Zn_3_, and AlLi, as well as a eutectic formed by Al(α) and Al_2_Mg_3_Zn_3_. In this investigation, the anode efficiency of the as-cast specimens was 61.43%. Taking into consideration this efficiency value, the authors inferred that the ternary alloy fulfilled the potential criteria established by the commercial manuals of anodes. Moreover, researchers asseverated that equiaxed grain presence favored diminution of the eutectic phase, and thus, the anode dissolution was more uniform and its efficiency was increased. 

Orozco et al. [11] studied the influence of Mg content on the electrochemical performance and efficiency of Al, with 5.3 at.% Zn and 5.3, 6.6, 7.2, and 11.3 at.% Mg applied as sacrifice anodes. The authors reported that the as-cast microstructure was composed of Al(α) dendrites, as well as eutectic (α + τ) and τ phases, which were both interdendritic. In this case, the calculated electrochemical efficiency was 68% for the sample with 5.3% Mg and 75% for 11.3% Mg addition.

The electrochemical techniques employed for corrosion research are polarization, linear polarization and voltammetry, which are all based on direct current [12]. In addition, electrochemical impedance spectroscopy (EIS) [12] and electrochemical frequency modulation [13] depend on alternating current. All techniques have advantages and drawbacks. For example, EIS can disclose different corrosion phenomena in one sample at various times [14]. However, EIS results analysis can be very difficult [14].

The simplest impedance analysis is electric analogs, but they have limitations on their physical interpretation, such as the geometry sense in porous systems, diffusion phenomena in irregular geometries, and adsorption of chemical intermediates in a corrosion process [14]. Hence, other methods should be applied, for instance, neuronal networks, transmission lines, and chemical mechanisms [14].

Kendamm applied an analysis of chemical adsorption to Fe in acid solution [15], MacDonald employed it to aluminum in basic solution [16], and Epelboin used it on nickel and cobalt in acid electrolyte [17]. This kind of impedance evaluation is not spread out among electrochemical researchers. This is due to the need for mathematical concepts of electrical engineering, which are not common among chemists [14]. 

Previous research was focused on ternary alloys (Al-Zn-activator element) due to intermetallic ternary compounds (Al_x_Zn_y_X_z_) promoting aluminum depassivation and decreasing the pitting corrosion effect. There are several ways to explain how a third alloying element modifies the aluminum passivation, such as detachment of the passive film, diffusion of aluminum through the activator particles, and reversible redox reactions of the activator catalyzing the transfer of electrons [18]. However, most recent studies used the theory of detachment of the passive film. In addition, the metallurgical characterization of the intermetallic particles associated with their size, localization, and chemical composition using statical methods has not been done extensively, although the importance of these facts is well known. 

A binary Al-Zn alloy has not been studied as a principal material in research because Zn can activate the Al surface, but the reached current efficiency is low and the corrosion potential is high in comparison with Al-xZn-activator element alloys [5,6]. A precise description of Zn’s role in the Al activation is not explicitly declared in any information source. Hence, fundamental studies of these topics are required.

Electrochemical impedance spectroscopy was applied to study sacrificial anodes. However, the main analysis tool is the equivalent analog (equivalent circuit), which is very restricted in electrochemical scopes and concepts. The basic mathematical frameworks of different circuit elements are not well understood and they are applied mistakenly. Reaction mechanisms based on the models of Kendal [15] and Epelboin [17] are a step forward toward improving the EIS results analysis because specific reactions are involved in the explanations of the anode degradation. 

Since most studies focused on the corrosion performance of Al-based alloys to be applied in cathodic protection used ternary Al-based alloys, the present research studied a simpler binary Al-Zn alloy system for these purposes, which will serve as a reference basis for cathodic protection studies. Furthermore, most efforts focused on studying the cathodic protection performance of Al-based alloys employed conventional electrochemical techniques, such as EIS; however, this technique has the abovementioned limitations. As such, when it is necessary to estimate parameters of corrosion mechanism, such as reaction constant rates, mathematical model analysis is useful. In this sense, a novel contribution of the present work was to develop a study of the corrosion performance of binary Al-xZn alloys by using the EIS technique complemented with a mathematical model that was related to an adsorption electrochemical mechanism.

For this reason, in the present work, the incidence of Zn content added to Al on its corrosion performance was analyzed. Based on previous works, it was expected that Zn addition to Al will promote a change in its corrosion mechanism.

In this context, the aim of this work was to characterize the corrosion behavior of Al alloyed with Zn (1.5, 3, and 5 at.%) in a 3 wt.% NaCl electrolyte. For this characterization, the electrochemical techniques applied were open circuit potential measurements, polarization curves, linear polarization, and electrochemical impedance spectroscopy. The results of the EIS were analyzed using electrochemical analogs and an adsorption reaction mechanism. 

## 2. Materials and Methods

**Figure 1:**
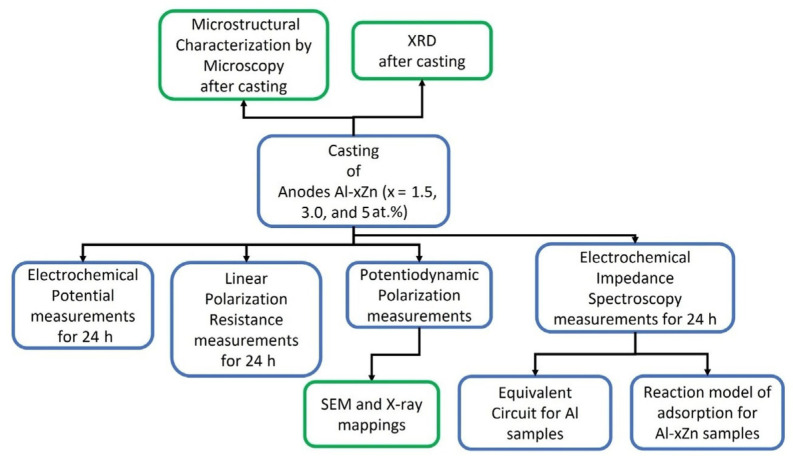
Process flow diagram that involves the experimental stages that involve the alloy production, their microstructural and electrochemical characterization, and an analysis of the EIS data.

Figure 1 shows a process flow diagram that consists of the experimental stages that involve the alloy AlxZn (x = 1.5, 3, and 5 at.%) production, as well as the microstructural and electrochemical characterization of the as-cast specimens, followed by a microstructural and chemical characterization of the corroded surfaces of the specimens, and finally, an analysis of the EIS data using equivalent circuits and a reaction model of adsorption for the Al-xZn samples.

### 2.1. Materials

Cast ingots of Al-xZn (x = 1.5, 3, and 5 at.%) alloys were created using an electrical resistance furnace at 800 °C. High-purity (99.9%) Al and Zn were placed in a SiC crucible to be melted. The alloys were cast in cylindrical ceramic molds with the aim to control the cooling rate to produce equiaxed dendrites. The approximate cooling rate that was estimated during solidification was 0.15 °C/s.

### 2.2. Electrochemical Corrosion Tests

The electrochemical corrosion evaluation was accomplished by using a Gamry Interphase 1000 potentiostat, whose manufacturer was in the Township of Warminster, PA, USA. The electrochemical cell was a three-electrode system. The reference electrode (RE) was a saturated calomel electrode (SCE), the counter electrode (CE) was a Pt wire, and Al-Zn alloys were the working electrodes (WE). The electrolyte was a solution of 3 wt.% NaCl at room temperature.

The open-circuit potential was measured for 24 h. Potentiodynamic polarization was recorded after 20 min, where the test was conducted from −400 mV to 900 mV vs. E_corr_ with a scan rate of 1 mV/s. The polarization resistance (LPR) was measured within an interval of ±20 mV vs. E_corr_ with a scan rate of 10 mV/min over 24 h. Impedance spectroscopy (EIS) experiments were recorded at an open-circuit potential; the applied frequency range was from 100,000 to 0.01 Hz with a perturbation of ±10 mV.

### 2.3. Microstructural Characterization

The microstructures of as-cast samples were characterized using optical microscopy in a Zeiss Microscope; the headquarters where these microscopes were manufactured was in Jena, Germany. The as-cast specimens were characterized using X-ray diffraction (XRD) analysis in a Bruker© diffractometer model D8 Advance. The headquarters of Bruker is in Billerica, MA, USA. The energy source had Kα line radiation with λ = 0.15406 nm. The 2ϴ range was from 10° to 100° with a step size of 0.02° and time per step of 0.6 s. The morphology and elements distribution on the alloy surfaces were analyzed using a scanning electron microscopy (SEM) and X-ray energy dispersive (EDS) analyzer. The equipment was a JEOL model JSM IT 500 SEM. The manufacturer of the JEOL equipment was in Peabody, MA, USA. 

### 2.4. Software Methods

The fitting process of the impedance results for the impedance transfer function associated with the electrochemical mechanism followed five steps that involved applying a genetic algorithm, evolution strategies, and the Levenberg–Marquardt method. The tool used for the fitting process was Ellis2, which is a software package developed for the optimization of complex functions of one variable. Ellis relies on Igor Pro^®^, which is a wave-based data analysis system, and gencurvefit, which is an implementation of a different evolution algorithm that was originally developed to fit neutron scattering data.

The fitting process of the impedance results for the impedance transfer function associated with the electrochemical mechanism followed five steps [19]: (1) Defining a space based upon lower and upper limits. (2) The model generates a random population of solutions. (3) The model tests all the plausible solutions and ranks them according to the error. (4) The solution with the highest error “dies” and is replaced by a new random vector of solutions. (5) The remaining members of the population exchange information to generate new solutions. 

The optimization was done using differential evolution, which is a hybrid between two methods genetic algorithm and evolutionary strategies. Differential evolution allows for finding a general minimum instead of local minima of the error function [20].

The informatics tool used for the fitting process was Ellis2 [21], which is a software package developed for the optimization of complex functions of one variable. Ellis2 relies on Igor Pro^®^, which is a wave-based data analysis system, and gencurvefit [22], which is an implementation of a different evolution algorithm that was originally developed to fit neutron scattering data. The errors in all the fittings were less than 10%. 

## 3. Results

### 3.1. Microstructural Analysis

**Figure 2:**
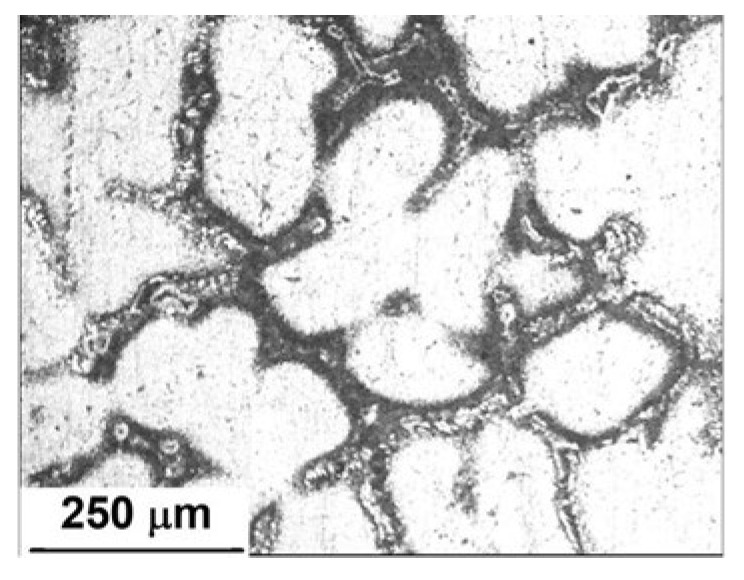
Microstructure of an as-cast Al-5%Zn alloy.

Figure 2 shows the microstructures of an Al-5%Zn alloy. It was composed of a white Al-rich matrix phase with a morphology of equiaxial dendrites and an interconnected secondary Zn-rich phase around the Al matrix. It is well known that Zn is precipitated in grain boundaries or interdendritic regions of Al-Zn alloys [9]. In accordance with the Al-Zn phase diagram [23], the solid solubility of Zn in Al is less than 1.2 at.% at room temperature. If the Zn content is higher, two solid solutions of zinc and Al are precipitated [24].

**Figure 3:**
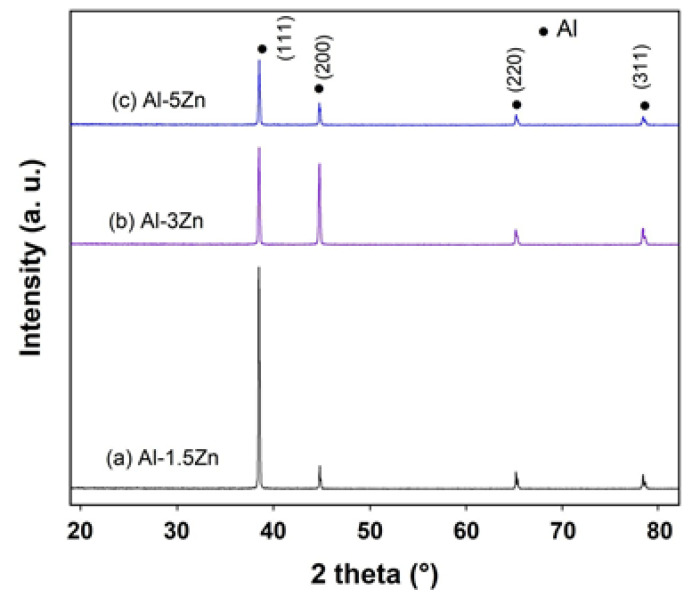
X-ray diffraction patterns of as-cast Al-xZn alloys.

Figure 3 exhibits the X-ray diffraction profiles of as-cast Al-xZn alloys (x = 1.5, 3, and 5 at.%). The diffractograms show that all the diffraction peaks corresponded to the Al element, with their respective (111), (200), (220), and (311) Miller indexes. The addition of Zn to Al did not modify the crystal structure of Al; however, the lattice constant of Al was modified by the solid solution of Zn inside the crystal lattice. Moreover, there was no evidence of any additional peaks belonging to Zn or Al_x_Zn_y_ phases. The ratio of intensities I_(111)_/I_(200)_ was found to be significantly different for each alloy associated with different crystallographic orientations between the materials. 

### 3.2. Potentiodynamic Polarization Curves

**Figure 4:**
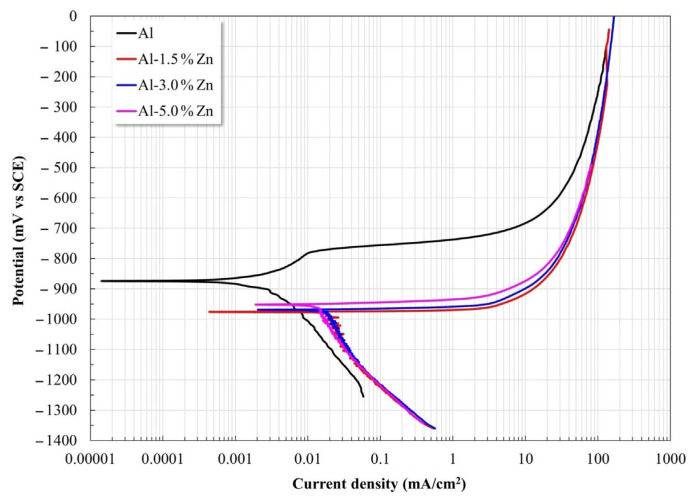
Potentiodynamic polarization curves of Al-xZn alloys exposed to an aqueous NaCl electrolyte.

Polarization curves for all the materials are shown in Figure 4. The Al samples displayed a slight pseudo-passivation process at low overpotential, while the samples with Zn exhibited a predominant anodic dissolution without the presence of an observable passivation process. The Zn additions induced a shift of E_corr_ toward the active side and increased the corrosion current density I_corr_ (Table 1). This behavior was reasonable since it was reported that additions of small amounts of Mg, Zn, Hg, and others lead to Al becoming more active [25]. 

The Al sample showed a pitting process after the pseudo-passivation regime, where disruption of the passive state was due to the interaction between the Al(OH)_3_ film and the Cl^−^ ions, which can lead to the formation of AlCl_3_ after enough time. Subsequently, AlCl_3_ dissolves as [AlCl_4_^−^] at higher values than the pitting potential [26,27,28]. Uniform corrosion of Al allows for the formation of Al_2_O_3_. However, Al_2_O_3_ is not stable in water, which leads to Al(OH)_3_ being produced via hydration.

The kinetic parameters determined from the polarization curves are presented in Table 1. The anodic slope decreased as the Zn content rose, which was associated with a progressive increase in the dissolution rate. Furthermore, the Zn additions induced a combined effect: the corrosion potential shifted to the electronegative side together with an increase in the corrosion current densities (I_corr_).

### 3.3. Open Circuit Potential

**Figure 5:**
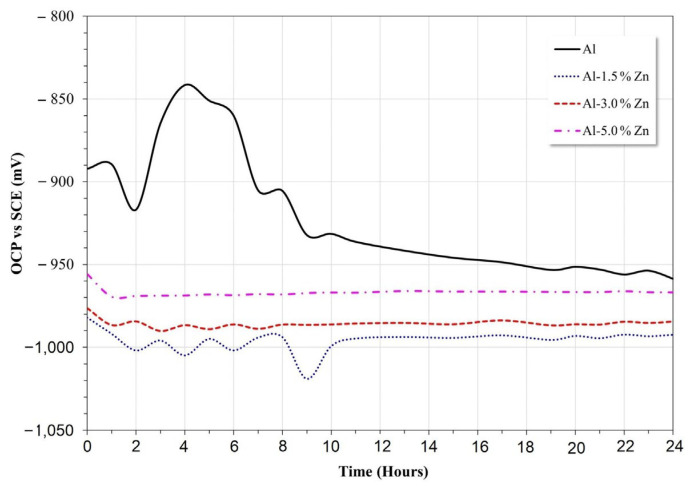
Open circuit potential vs. time of Al-xZn alloys in aqueous NaCl.

The results of the open circuit potential for all materials are shown in Figure 5. In the early stage, the potential of pure Al changed to the anodic direction between 2 h and 4 h of immersion, then the potential decreased until 16 h, and after that, the potential remained quasi-stable. This behavior was associated with the formation of Al_2_O_3_ and Al(OH)_3_ according to the Pourbaix diagram [29].

The Zn additions modified the behavior of the passive nature of the Al electrode. The Al-xZn alloys exhibited greater electronegative potentials than the pure aluminum electrode. The Al-5%Zn, Al-3%Zn, and Al-1.5%Zn alloys exhibited average open-circuit potentials of 0.96 V, 0.98 V, and 0.99, respectively. The active behavior may be related to the decrease in pH value due to the formation of Zn(OH)_2_, as indicated in the Pourbaix diagram [29] of Zn in neutral H_2_O, where it can be observed that on initial exposure of Zn, the formation of Zn(OH)_3_^−^ complexes was promoted by reactions presented in Equations (1) and (2):

(1) $${\mathrm{Zn}}_{\left(s\right)}+{\mathrm{OH}}^{-}⇄\mathrm{Zn}{\left(\mathrm{OH}\right)}_{\left(\mathrm{ads}\right)}+e^{-}$$

(2) $$\mathrm{Zn}{\left(\mathrm{OH}\right)}_{\left(\mathrm{ad}\right)}+{\mathrm{OH}}_{\left(\mathrm{aq}\right)}^{-}⇄\mathrm{Zn}{\left(\mathrm{OH}\right)}_{2\left(\mathrm{ad}\right)}+e^{-}$$

In the presence of NaCl, the film becomes porous via chloride penetration and induces the formation of Zn_5_(OH)_8_Cl_2_ 2H_2_O, in agreement with the following set of reactions [30]:

(1) The cathodic reaction corresponds to oxygen reduction:
  (3) $$O_{2\left(g\right)}+H_{2}O_{\left(l\right)}+4e^{-}\to 4{\mathrm{OH}}_{\left(\mathrm{aq}\right)}^{-}$$
(1) The anodic reaction implicates the dissolution of Zn:
  (4) $$\mathrm{Zn}\to Zn^{2+}+2e^{-}$$

Zinc and hydroxide anions interact to form zinc hydroxide, as noted in reactions (1) and (2). The pH could be sufficiently high at active cathodic sites in order to form $\mathrm{Zn}{\left(\mathrm{OH}\right)}_{4}^{2-}$, in accordance with the following reaction [30]:

(5) $$\mathrm{Zn}{\left(\mathrm{OH}\right)}_{2\left(s\right)}+2{\mathrm{OH}}_{\left(\mathrm{aq}\right)}^{-}⇄\mathrm{Zn}{\left(\mathrm{OH}\right)}_{4\left(\mathrm{aq}\right)}^{-}$$

With the presence of NaCl, the Cl^−^ ions move to anodic sites where zinc hydroxide chloride is produced in accordance with the following reaction [30]:

(6) $$5\mathrm{Zn}{\left(\mathrm{OH}\right)}_{2\left(s\right)}+2{\mathrm{Cl}}^{-}+H_{2}O\to {\mathrm{Zn}}_{5}{\left(\mathrm{OH}\right)}_{8}{\mathrm{Cl}}_{2}+2{\mathrm{OH}}_{\left(\mathrm{aq}\right)}^{-}$$

The potential transient during the first 10 h disappeared with the Zn additions, as shown in Figure 5. In this case, the oxide layer formed on the surface of the Al alloy was typically porous and allowed for Cl^−^ ion penetration where the localized failure of the passive layer gave rise to the initiation of pits. Some pits could be susceptible to the repassivation process and others gave rise to the formation of the AlCl_4_^−^ complex [2]. Alloying elements, such as Zn or In, are added to Al in order not only to shift its operating potential (pitting potential) toward sufficiently electronegative values but also to induce a more uniform corrosion morphology [31]; this was consistent with the disappearance of transients as the Zn concentration increased. 

### 3.4. Linear Polarization Resistance

**Figure 6:**
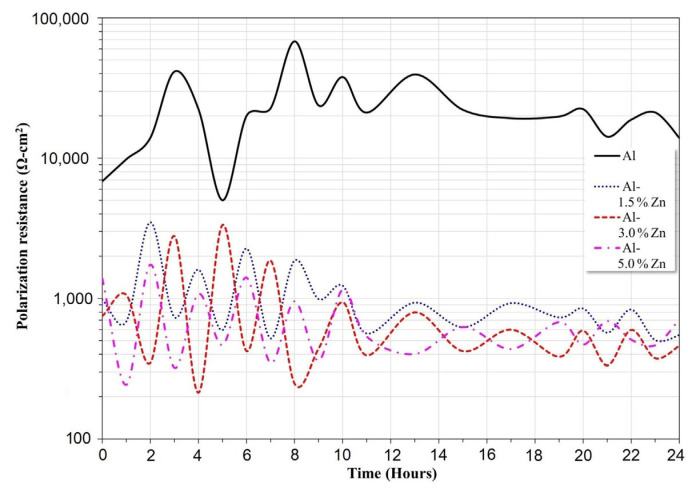
Variation of polarization resistance (R_p_) of Al-xZn alloys as a function of the exposure period in a solution of 3 wt.% NaCl.

The variation of R_p_ as a function of time for all the materials is presented in Figure 6. The Al sample exhibited the highest R_p_ values throughout the exposure period. This behavior was associated with a minor corrosion rate, which was expressed in agreement with the Stern–Geary equation [32]. The polarization resistance decreased as the Zn concentration went up; this trend was attributed to an increase in the corrosion rate. In this case, the R_p_ mean values of Al-1.5Zn, Al-3Zn, and Al-5Zn were 1049, 825, and 712 Ohm.cm^2^, respectively. The purpose of adding elements such as Ti, Ga, Zn or Sn [4,33] is to remove the passivation film of Al_2_O_3_, producing an increase in the corrosion rate, which is associated with a decrease in R_p_ values. It is worth noticing that the R_p_ of Al-xZn alloys exhibited lower fluctuations than pure Al. This behavior was related to the protective film on Al undergoing passivation, pitting, and a repassivation process, while Al-Zn experienced corrosion, partial passivation, and detachment.

### 3.5. Electrochemical Impedance Spectroscopy

**Figure 7:**
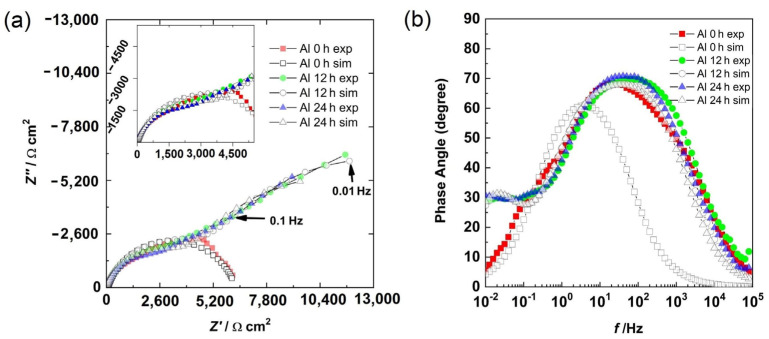
Results for aluminum at different times: (**a**) Nyquist diagram and (**b**) Bode diagram.

The Bode phase angle and Nyquist diagrams for the Al sample are shown in Figure 7. At 0 h, the presence of a semicircle (Figure 7a) and a well-defined time constant between the high and medium frequency region (Figure 7b) were observed. This could be associated with the capacitive response of the surface. However, at 12 h and 24 h of immersion, in addition to the high- and medium-frequency semicircles, the beginning of another semicircle in the low-frequency region was observed (Figure 7a). From the Bode diagram (Figure 7b), in the low-frequency region, the presence of a time constant (phase angle around 30 degrees) was observed, and in the intermediate-frequency region, a second time constant with a phase angle greater than that of 0 h was observed. The first time constant could be associated with the formation of a layer of corrosion products, while the second time constant was associated with the capacitive response of the surface.

**Figure 8:**
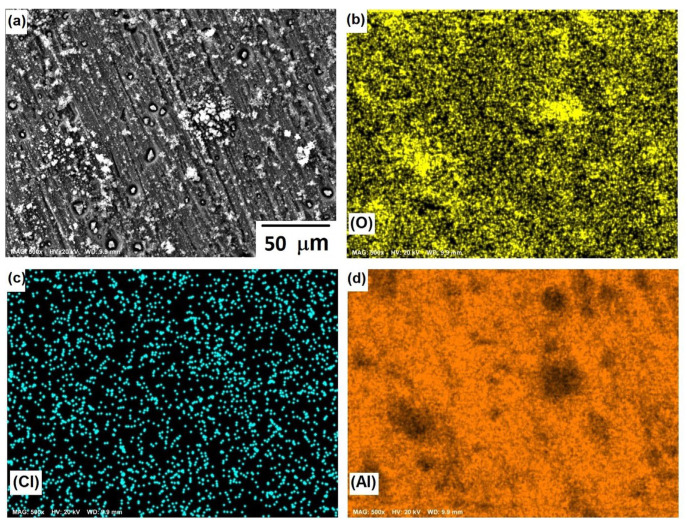
SEM micrographs of the corroded surface of (**a**) Al, and X-ray mappings of (**b**) O, (**c**) Cl, and (**d**) Al. All micrographs were obtained at 500× magnification.

Figure 8 shows the morphological aspects of Al and the element mapping after the corrosion test. The presence of a pitting attack was observed, and according to the mapping of elements, the formation of a thin layer of Al-based corrosion products (oxides or oxy-hydroxides). The presence of chlorine can be associated with both NaCl and Cl^−^ ions adsorbed onto a passive film since analytical techniques, such as SIMS and XPS [34], have shown the adsorption of Cl^−^ ions on protective films of Al. The micrograph displayed in Figure 8a shows solid particles inside pits, where the dissolution product AlCl_x_ gave rise to the formation of a solid salt; this was consistent with the results of previous research [35] and a salt film could stabilize some pits.

EIS is not suitable when a corrosion system is under pitting because the corrosion potential shifts quickly. This leads to completely noisy results. However, the impedance results were stable in this experiment. EIS can be applied when metastable pitting is occurring in an electrochemical system, just as in this experiment. Pitting is associated with chloride in the electrolyte due to the formation of complex compounds, which catalyze the local damage, but metastable pitting is not as fast as a pure pitting process. 

The semicircle at an HF was associated with the aluminum oxide layer and the LF was related to the charge transfer resistance in metastable pits and at places where uniform corrosion was occurring. At 0 h, the electrolyte was in contact with the Al matrix and the corrosion had initiated, and thus, a semicircle was observed. The results at 12 h and 24 h were very similar because the electrolyte had filled the pores, reaching the substrate, and metastable pitting had started. 

**Figure 9:**
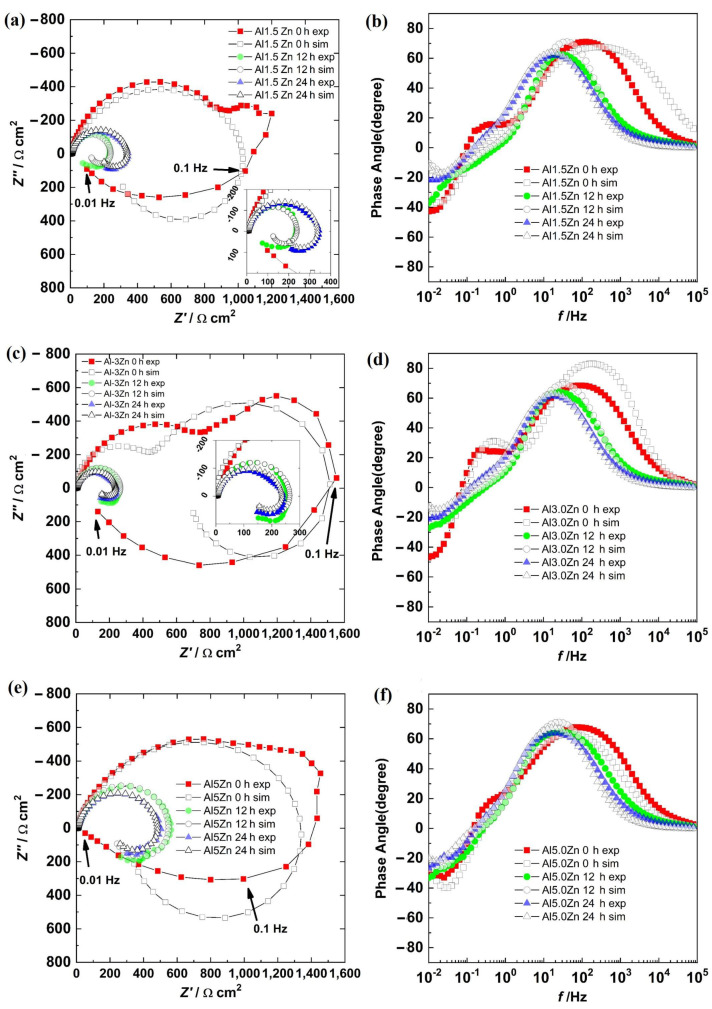
Nyquist diagrams: (**a**) Al-1.5Zn, (**c**) Al-3.0Zn, and (**e**) Al-5.0Zn. Bode diagrams: (**b**) Al-1.5Zn, (**d**) Al-3.0Zn, and (**f**) Al-5Zn.

The Nyquist and Bode phase angle diagrams for all the Al-Zn samples are displayed in Figure 9. The results showed a semicircle in the first quadrant and another semicircle in the fourth one for all the alloys. This was supported by the Bode diagram, where in the HF–MF range, the angles were positive, and at a LF, the angles were negative. This behavior was due to a corrosion process where a product layer was not presented and the adsorption of intermediate compounds was occurring. This behavior was different from the results of the Al sample. The additions of Zn could activate the corrosion of the aluminum matrix. However, surface SEM-EDS analyses showed no evidence of pitting corrosion and the corrosion products were not dense. This supported the EIS behaviors, where metastable pitting is not occurring. The addition of Zn promoted uniform corrosion.

There were three semicircles in the Nyquist diagram for all the samples at 0 h. The first semicircle was in the HF–MF range, a second loop was at 1–0.1 Hz, and a third one was at 0.1–0.01 Hz. The two loops in the first quadrant were associated with the presence of corrosion products layer and an electrochemical reaction, while the third loop in the fourth quadrant was related to the adsorption of the chloride intermediates. The second semicircle tended to disappear with time as the Zn content increased.

The results for all the samples in the Nyquist diagram at 12 h and 24 h described two semicircles associated with a charge transfer process with the adsorption of intermediates. Impedance values decreased from 0 h to 12 h and then increased slightly at 24 h. 

**Figure 10:**
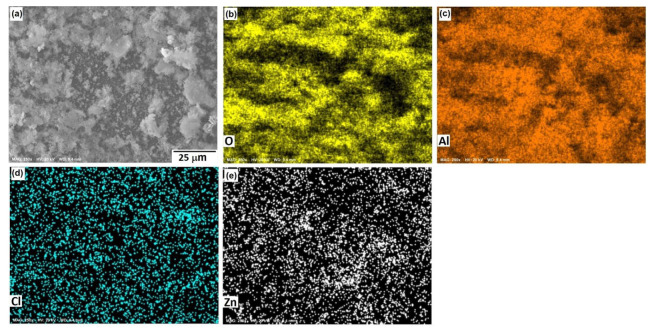
SEM micrographs of the corroded surface of (**a**) the Al-5%Zn alloy with the elemental mapping of (**b**) O, (**c**) Al, (**d**) Cl, and (**e**) Zn. All micrographs were obtained at 250× magnification.

**Figure 11:**
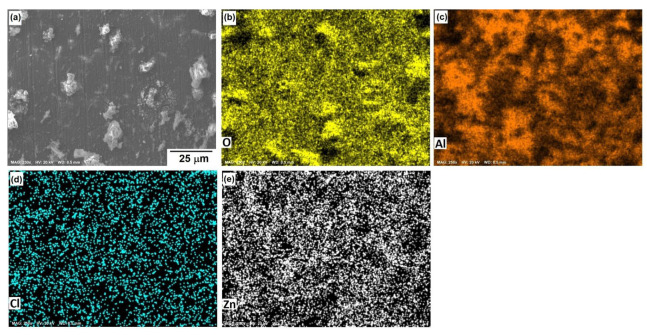
SEM micrographs of the corroded surface of (**a**) the Al-3%Zn alloy with the elemental mapping of (**b**) O, (**c**) Al, (**d**) Cl, and (**e**) Zn. All micrographs were obtained at 250× magnification.

**Figure 12:**
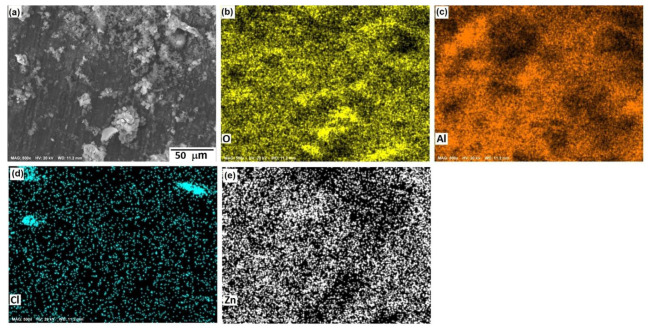
SEM micrographs of the corroded surface of (**a**) the Al-1.5%Zn alloy with the elemental mapping of (**b**) O, (**c**) Al, (**d**) Cl, and (**e**) Zn. All micrographs were obtained at 500× magnification.

Figure 10, Figure 11 and Figure 12 show the morphological aspects of the Al-Zn alloys and the element mapping after the corrosion test. In all cases, the same characteristics were observed. The mapping of Al and O demonstrated the presence of Al-oxide. The formation of this protective layer of aluminum oxide was reported in multiple research papers [4,5]. The presence of Al and Cl could be associated with AlCl_3_ [36,37], which is formed by the interaction between an Al(OH)_3_ film and Cl^−^ ions, which occurs after the breakdown of the passive state of Al in a saline medium. The presence of Zn, Cl, and O could be related to the formation of a non-protective zincate film ZnOZnCl_2_ [34], which was formed by the interaction of Zn(OH)_2_ or ZnO passive films with Cl^−^ [32]. It is worth noticing that for longer exposure times, the products formed on Zn included Zn_5_(OH)_8_Cl_2_.H_2_O [27,30] and some other corrosion products composed mainly of Al, Zn, Cl, and O [38]. 

### 3.6. Impedance Spectroscopy Modeling

The equivalent analog that represents electrochemical reactions at a porous interface [39] is a parallel combination of the reaction resistance (*R_ct_*) and the pseudocapacitance of the double layer (*Q_dl_*) of a phase constant element. This last element combination is in series with the film resistance (*R_film_*), and all these components are in parallel with the film pseudocapacitance (*Q_dl_*) of a phase constant element [39,40]. In addition, the solution resistance (*R_s_*) is in series with the rest of the elements. The mathematical expression is shown in Equation (7).

(7) $$Z_{T0}\left(j\omega \right)=R_{s}+\frac{1}{\frac{1}{R_{film}+\frac{1}{R_{ct}}+Q_{dl}{\left(j\omega \right)}^{n_{dl}}}+Q_{film}{\left(j\omega \right)}^{n_{film}}}$$

where *j* is the imaginary number, ω is the angular frequency, *n_dl_* is the exponent of the phase constant element of the double layer, and *n_film_* is the exponent of the phase constant element of the film. 

A mechanism of adsorption was proposed by Epelboin [17]. This model was applied to passivation behaviors, where adsorbed chemical groups are an important part of the corrosion process. An adaptation of this model was used in this work and it is described below.

The corrosion process of Al-Zn should include oxidation reactions of Al and Zn, but the complexity of the adsorption model increases considerably [41]. Hence, the zinc reactions are not considered due to the role of the element. The added Zn can develop defects in the interface due to the different phases present in the alloy, thereby causing cracking and detachment of the alumina layer formed [42]. In addition, the activator (Zn) could inhibit the aluminum oxide formation that results for different reasons, such as the diffusion of aluminum ions through activator particles, lowering the electronic and/or conductivity properties of the corrosion product film, reversible reactions of the activator, and biasing of the electrode surface charge to facilitate the adsorption of aggressive ions at the surface [42].

The reaction of aluminum in a solution of chloride is based on the following reactions [26]:

(8) $${\mathrm{Al}}_{\left(s\right)}+3{\mathrm{OH}}_{\left(\mathrm{aq}\right)}^{-}\overset{\;k_{1}\;}{\to }\mathrm{Al}{\left(\mathrm{OH}\right)}_{3\left(s\right)}+3e$$

(9) $$\mathrm{Al}{\left(\mathrm{OH}\right)}_{3\left(s\right)}+{\mathrm{Cl}}_{\left(\mathrm{aq}\right)}^{-}\overset{\;k_{2}\;}{\to }\mathrm{Al}{\left(\mathrm{OH}\right)}_{2}{\mathrm{Cl}}_{\left(\mathrm{ad}\right)}+{\mathrm{OH}}_{\left(\mathrm{aq}\right)}^{-}$$

(10) $$\mathrm{Al}{\left(\mathrm{OH}\right)}_{2}{\mathrm{Cl}}_{\left(\mathrm{ad}\right)}+{\mathrm{Cl}}_{\left(\mathrm{aq}\right)}^{-}\overset{\;k_{3}\;}{\to }\mathrm{Al}\left(\mathrm{OH}\right){\mathrm{Cl}}_{2\left(\mathrm{ad}\right)}+{\mathrm{OH}}_{\left(\mathrm{aq}\right)}^{-}$$

(11) $$\mathrm{Al}\left(\mathrm{OH}\right){\mathrm{Cl}}_{2}{}_{\left(\mathrm{aq}\right)}+{\mathrm{Cl}}_{\left(\mathrm{aq}\right)}^{-}\overset{\;k_{4}\;}{\to }{\mathrm{AlCl}}_{3\left(\mathrm{aq}\right)}+{\mathrm{OH}}_{\left(\mathrm{aq}\right)}^{-}+\mathrm{ss}$$

(12) $$\mathrm{ss}+O_{2\left(\mathrm{aq}\right)}+4H_{\left(\mathrm{aq}\right)}^{+}+4e^{-}\overset{\;k_{5}\;}{\to }2H_{2}O_{\left(l\right)}$$

Here, “*ss”* means a surface site. The expression of the faradaic current is shown in Equation (13), while the relationship between the coverage factor and potential can be found from the dependency of the coverage with time in non-steady conditions (Equations (14)–(17)) [43].

(13) $$\frac{i_{f}}{F}=[3k_{1}\left(1-\theta_{1}-\theta_{2}-\theta_{3}-\theta_{4}\right)[{\mathrm{OH}}^{-}]-4k_{5}\theta_{4}[O_{2\left(\mathrm{aq}\right)}]\left[H^{+}\right]]$$

(14) $$\beta_{1}\frac{d\theta_{1}}{dt}=3k_{1}\left(1-\theta_{1}-\theta_{2}-\theta_{3}-\theta_{4}\right)\left[{\mathrm{OH}}^{-}\right]-k_{2}\theta_{1}\left[{\mathrm{Cl}}^{-}\right]$$

(15) $$\beta_{2}\frac{d\theta_{2}}{dt}=k_{2}\theta_{1}\left[{\mathrm{Cl}}^{-}\right]-k_{3}\theta_{2}\left[{\mathrm{Cl}}^{-}\right]$$

(16) $$\beta_{3}\frac{d\theta_{3}}{dt}=k_{3}\theta_{2}\left[{\mathrm{Cl}}^{-}\right]-k_{4}\theta_{3}\left[{\mathrm{Cl}}^{-}\right]$$

(17) $$\beta_{4}\frac{d\theta_{4}}{dt}=k_{4}\theta_{3}\left[{\mathrm{Cl}}^{-}\right]-4k_{5}\theta_{4}\left[O_{2\left(\mathrm{aq}\right)}\right]\left[H^{+}\right]$$

Here, $K_{1}=K_{1}^{0}e^{bE}$ and $k_{5}=k_{5}^{o}e^{-bE}$; *i_F_* is the faradaic current; *F* is the Faraday constant; *b_i_* is the inverse of the cathodic or anodic slope of the Tafel behavior; *E* is the potential vs. *E_ocp_*; *k_i_* is the rate constant of reaction; *θ_i_* is the coverage factor associated with each intermediary adsorbed at the electrode surface; *β_i_* is a constant that links the coverage fraction with the surface concentration; [*X*] is the concentration of a reactant or product; and *X* refers to either chloride, hydroxide, or hydronium ions, or dissolved oxygen.

The steady-state expression of Equations (14)–(17) are shown in the expressions (18)–(21), respectively.

(18) $$\bar{\theta_{1}}=\frac{12\left[H^{+}\right]\left[O_{2\left(\mathrm{aq}\right)}\right]\left[{\mathrm{OH}}^{-}\right]k_{3}k_{4}k_{1}k_{5}}{X}$$

(19) $$\bar{\theta_{2}}=\frac{12\left[H^{+}\right]\left[O_{2\left(\mathrm{aq}\right)}\right]\left[{\mathrm{OH}}^{-}\right]k_{2}k_{4}k_{1}k_{5}}{X}$$

(20) $$\bar{\theta_{3}}=\frac{12\left[H^{+}\right]\left[O_{2\left(\mathrm{aq}\right)}\right]\left[{\mathrm{OH}}^{-}\right]k_{2}k_{3}k_{1}k_{5}}{X}$$

(21) $$\bar{\theta_{4}}=\frac{3\left[{\mathrm{Cl}}^{-}\right]\left[{\mathrm{OH}}^{-}\right]k_{2}k_{3}k_{4}k_{1}}{X}$$

(22) $$X=12\left[{\mathrm{OH}}^{-}\right]\left(\left[H^{+}\right]\left(\left(k_{2}+k_{4}\right)k_{3}+k_{2}k_{4}\right)\left[O_{2\left(\mathrm{aq}\right)}\right]k_{5}+\frac{1}{4}\left[{\mathrm{Cl}}^{-}\right]k_{2}k_{3}k_{4}\right)k_{1}+4\left[\mathrm{Cl}\right]\left[H\right]k_{5}\left[O\right]k_{2}k_{3}k_{4}$$

where $\bar{\theta_{i}}$ is the steady-state coverage factor associated with the intermediary *i* in the corrosion mechanism. 

The faradaic impedance *Z_f_* can be obtained by linearizing Equations (14) and (17) for a small sinusoidal wave perturbation of potential [41]. It is shown in the expressions (24) and (26).

(23) $$\beta_{1}j\omega \delta \theta_{1}-\left(-3k_{1}\left[{\mathrm{OH}}^{-}\right]-k_{2}\left[{\mathrm{Cl}}^{-}\right]\right)\delta \theta_{1}=\left(-3k_{1}b_{1}\left[{\mathrm{OH}}^{-}\right]\left(1-\bar{\theta_{1}}-\bar{\theta_{2}}-\bar{\theta_{3}}-\bar{\theta_{4}}\right)\right)\delta E$$

(24) $$\frac{\delta \theta_{1}}{\delta E}=\frac{\left(-3k_{1}b_{1}\left[{\mathrm{OH}}^{-}\right]\left(1-\bar{\theta_{1}}-\bar{\theta_{2}}-\bar{\theta_{3}}-\bar{\theta_{4}}\right)\right)}{\left(\beta_{1}j\omega +3k_{1}\left[{\mathrm{OH}}^{-}\right]+k_{2}\left[{\mathrm{Cl}}^{-}\right]\right)}$$

(25) $$\beta_{4}j\omega \delta \theta_{4}=\left(-4k_{5}\left[O_{2\left(\mathrm{ac}\right)}\right]\left[H^{+}\right]\right)\delta \theta_{4}+\left(4k_{5}b_{2}\theta_{4}\left[O_{2\left(\mathrm{aq}\right)}\right]\left[H^{+}\right]\right)\delta E$$

(26) $$\frac{\delta \theta_{4}}{\delta E}=\frac{\left(4k_{5}b_{2}\bar{\theta_{4}}\left[O_{2\left(\mathrm{aq}\right)}\right]\left[H^{+}\right]\right)}{\left(\beta_{4}j\omega +4k_{5}\left[O_{2\left(\mathrm{aq}\right)}\right]\left[H^{+}\right]\right)}$$

Here, *δθ* is the coverage variation from a small wave variation of potential, *δE* is the small wave perturbation of potential, *j* is the imaginary number, and ω is the angular frequency. 

The *Z_f_* relation is displayed in Equation (27) [41].

(27) $$\frac{1}{Z_{f}}=\frac{1}{R_{ct}}-F\left[\left(-3k_{1}\left[{\mathrm{OH}}^{-}\right]\right)\frac{\delta \theta_{1}}{\delta E}+\left(-3k_{1}\theta_{4}\left[OH\right]-4k_{5}\left[O_{2\left(\mathrm{aq}\right)}\right]\left[H^{+}\right]\right)\frac{\delta \theta_{4}}{\delta E}\right]$$

(28) $$\frac{1}{R_{ct}}=F\left[3k_{1}b_{1}\left(1-\bar{\theta_{1}}-\bar{\theta_{2}}-\bar{\theta_{3}}-\bar{\theta_{4}}\right)\left[OH^{-}]+4k_{5}b_{5}\bar{\theta_{5}}\left[O_{2\left(\mathrm{aq}\right)}\right][H^{+}\right]\right]$$

The total impedance *Z_T_* for 9 h and 12 h is shown in (29) [41].

(29) $$Z_{T}\left(j\omega \right)=R_{s}+\frac{1}{\frac{1}{Z_{f}\left(j\omega \right)}+Q_{dl}{\left(j\omega \right)}^{n_{dl}}}$$

where *Z_T_* is the total impedance of the interface, *R_s_* is the solution resistance, *Q_dl_* is the pseudocapacitance of the double layer, *n_dl_* is the exponent of the phase constant element, *j* is the imaginary number, and ω is the angular frequency.

The total impedance *Z_T2_* for the Al-Zn samples at 0 h and Al at 0 h is given in Equation (30) [40]:

(30) $$Z_{T_{2}}\left(j\omega \right)=R_{s}+\frac{1}{\frac{1}{R_{film}+\frac{1}{Z_{f}\left(j\omega \right)}+Q_{dl}{\left(j\omega \right)}^{n_{dl}}}+Q_{film}{\left(j\omega \right)}^{n_{film}}}$$

The quantification of the variables was done by fitting the experimental results with the proposed models. The fitting process followed five steps described previously, which involved applying a genetic algorithm, evolution strategies, and the Levenberg–Marquardt method [20]. In addition, the solutions were found within physically meaningful ranges of the variables. The graphical results are shown in Figure 6 and Figure 8, while the values of the parameters are displayed in Table 2 and Table 3. The experimental data and the model fitting results had good agreement, as seen in Figure 6 and Figure 8.

The parameters obtained for the Al samples are displayed in Table 2, while the results for the Al-xZn samples are shown in Table 3. The reaction constant *k*_1_ associated with reaction (8) for 0 h was 6081, which is a high value. The formation of aluminum hydroxide is important in the aluminum sample. On other hand, *k*_1_ was very low for the Al-Zn alloys, where its value was in the order of 10^−9^ to 10^−11^. The hydroxide formation was lowered due to Zn’s effect on the corrosion products film [42]. This did not mean a lack of alumina layer as seen in micrographics in Figure 9, Figure 10 and Figure 11. 

The reaction constants *k*_2_, *k*_3_, and *k*_4_ were in the order of thousands and were related to the dissolution of the alumina layer by the action of chloride. This phenomenon is called “depassivation due to Cl^−^ penetration” [44]. Reactions (9) to (13) showed the interaction of hydroxide, which was already formed; these transformations were fast, but they did not occur on all the surfaces. The formation of aluminum chloride could be linked with the mappings in Figure 9, Figure 10 and Figure 11. The constant *k*_5_ was related to the reduction of oxygen in reaction (14) and its order of magnitude was around 10^1^ to 10^3^, which is a high reaction rate. The reaction of oxygen was fast, but the cathodic reaction had a mixed control reaction–diffusion [11], as seen in Figure 3.

The cathodic and anodic constants *b_1_* and *b_2_*, respectively, had magnitudes between 3 V^−1^ and 8 V^−1^, which are the inverse of the Tafel slope corresponding to values between 333 mV/decade and 125 mV/decade. The fitting did not exactly reproduce the values recorded in the Tafel results due to the simplification in the reaction mechanism (e.g., the reactions of zinc were not considered). However, the results were in valid ranges. The *β*_1_ and *β*_2_ constants, which are relations between the coverage fraction and surface concentration, are in the order 10^−8^, as in [41]. 

The concentrations of hydroxide were generally 10^−10^ mol cm^−3^, which is a behavior related to pH = 7. On the other hand, the hydronium changed due to the interface features; this was the acidification of the corrosion process. The hydronium was linked to the pH modification called local thermodynamic equilibrium [45]. Hence, the hydroxide was linked to the bulk concentration, while the hydronium was associated with the local steady-state concentration [45].

The pseudocapacitance can be two orders of magnitude higher than the “real” capacitance [46]. The pseudocapacitances of the product film (*Q_film_*) were in the order of 10^−4^, which was two orders higher, in agreement with previous measurements [47]. Likewise, the pseudocapacitances of the double layer (*Q_dl_*) in chloride environments were in the order of 10^−6^, as in [11]. 

The set of results was in orders of magnitude that were consistent with previous experimental results. The main goal of the corrosion mechanism is knowing the reaction constant rate to expose some of the possible reactions that are occurring at the surface of the electrode. 

## 4. Conclusions

The addition of Zn increased the corrosion rate, where the addition of 1.5, 3.0, and 5 wt.% of Zn to Al induced a progressive diminution of the polarization resistance. Al exhibited a pseudo-passivation process during the potentiodynamic polarization test. On the other hand, the Al-Zn alloys underwent a corrosion process without the formation of protective compounds at the interface. This was reasonable since elements such as Zn, Mg, and Hg, are added to pure aluminum for cathodic protection applications.

The EIS results were consistent with the results of R_p_ and the polarization curves. The corrosion resistance of the Al-Zn alloys was lower than the Al material. The EIS analysis result that is found by applying a corrosion mechanism is a method that can provide a better understanding of the process than an electrical equivalent circuit. 

A contribution derived from applying the mathematical model to generate fitted curves to the EIS data revealed that the inductive behavior of these results was associated with the adsorption of chloride and oxygen at the surface. This interpretation made more sense than applying an inductor of an equivalent circuit, which has no real comparison at an electrochemical interface. 

The reaction mechanism showed that the reaction rate for hydroxide production is in the order of 10^3^ for aluminum, while it is the order of 10^−9^–10^−11^ for Al-Zn alloys. The inductive behavior of the Al-Zn was associated with the chloride and oxygen adsorption at the surface.

In general, the three alloys Al-xZn (x = 1.5, 3, and 5 at.%) assessed in the present work presented higher corrosion rates than those of ternary alloys, such as Al-5Zn-0.1Sn and Al-5Zn-0.2Sn, studied under the same concentration of electrolyte. Even the corrosion rates expressed in terms of i_corr_ determined in the three alloys evaluated in the present research were found to be higher than that of quaternary alloys of the system Al-Zn-In, Al-Zn-In, and Al-Zn-In.

Among some of the limitations of the present work, we can mention that more detailed analyses of the corrosion surface in terms of an exhaustive chemical and microstructural characterization were omitted. Furthermore, there was a lack of resources to perform an electrochemical efficiency assessment. Thus, in this sense, it will be very valuable to perform an exhaustive characterization of a corrosion surface in as-cast and corroded specimens using techniques such as XPS, Raman, and AES. Moreover, we propose to evaluate the electrochemical efficiency in future research to compare the derived results with the standard requirements for the sacrificial anodes.

Furthermore, in future works, the development of statistical data analysis of the model, such as error, sensitivity, and uncertainty analyses, is proposed to increase the quality of research.

## Figures and Tables

**Table 1:** Electrochemical parameters of Al-Zn base alloys exposed to 3 wt.% NaCl in water.

Material	E_corr_ (mV)	b_a_ (mV/Dec)	b_c_ (mV/Dec)	I_corr_ (mA/cm^2^)
**Al**	−874	192	342	0.00467
**Al-1.5%Zn**	−980	36	400	0.01991
**Al-3.0%Zn**	−970	36	366	0.01919
**Al-5.0%Zn**	−952	16	331	0.01413

**Table 2:** Fitting results of the model for Al.

	Time		
Parameter	0 h	12 h	24 h
***k*** **_1_**	6081	--	--
***k*** **_2_**	2359	--	--
***k*** **_3_**	6139	--	--
***k*** **_4_**	3738	--	--
***k*** **_5_**	4544	--	--
***b*** **_1_**	5	--	--
***b*** **_2_**	3	--	--
**OH**	1 × 10^−10^	--	--
**Cl**	0.008	--	-
**AlCl**	8.77 × 10^−7^	--	--
**H**	1 × 10^−10^	--	--
**O**	2.08 × 10^−5^	--	--
***β*** **_1_**	3.72 × 10^−8^	--	--
***β*** **_2_**	6.78 × 10^−8^	--	--
***R_s_***	69.54	7	7
***R_CT_***	5500	15,712	13,519
***Q_dl_***	6.36 × 10^−6^	6.53 × 10^−4^	8.41 × 10^−4^
***n_dl_***	0.99	0.8	0.8
***R_film_***	6222.5	5861	4773
***Q_film_***	1.19 × 10^−4^	6.24 × 10^−5^	7.82 × 10^−5^
***n_film_***	0.8	0.8	0.8

*k*_1_ (mol cm^−2^ s^−1^), *k*_2_ (cm s^−1^), *k*_3_ (cm s^−1^), *k*_4_ (cm s^−1^), *k*_5_ (mol cm^−2^ s^−1^), *b*_1_ (V^−1^), *b*_2_ (V^−1^), OH (mol cm^−3^), Cl (mol cm^−3^), AlCl (mol cm^−3^), H (mol cm^−3^), *O* (mol cm^−3^), *β*_1_ (mol cm^−2^), *β*_1_ (mol cm^−2^), *R_s_* (Ohm cm^−2^), *R_CT_* (Ohm cm^−2^), *Q_dl_* (F cm^−2^ s^n−1^), *R_film_* (Ohm cm^−2^), *Q_film_* (F cm^−2^ s^n−1^).

**Table 3:** Fitting results of the model for Al-xZn (x = 1.5, 3.0, and 5.0) alloys.

	Al-1.5%Zn			Al-3.0%Zn			Al-5.0%Zn		
	Time			Time			Time		
Parameter	0 h	12 h	24 h	0 h	12 h	24 h	0 h	12 h	24 h
***k*** **_1_**	1.79 × 10^−11^	1.39 × 10^−10^	1.44 × 10^−9^	1.88 × 10^−11^	9.97 × 10^−11^	8.28 × 10^−10^	2.72 × 10^−11^	2.72 × 10^−10^	1.32 × 10^−11^
***k*** **_2_**	6660	5601	9180	5264	423	828	7125	704	759
***k*** **_3_**	81,501	1044	7284	8331	29	983	3024	402	435
***k*** **_4_**	1982	3516	5537	5510	670	237	8045	710	531
***k*** **_5_**	---	1372	56	303	250	56	176	81	65
***b*** **_1_**	5.92	5	5	6	5.38	4.76	6	6	6
***b*** **_2_**	7.67	3	3	8	4	4	8	4	4
**OH**	1 × 10^−10^	1 × 10^−10^	1 × 10^−10^	1 × 10^−10^	3.13 × 10^−10^	1.67 × 10^−10^	1 × 10^−10^	1.70 × 10^−10^	3.54 × 10^−9^
**Cl**	0.004	0.004	0.004	0.002	0.005	0.003	0.008	0.006	0.005
**AlCl**	7.10 × 10^−7^	6.03 × 10^−7^	4.66 × 10^−7^	6.42 × 10^−7^	3.32 × 10^−7^	8.30 × 10^−7^	9.89 × 10^−7^	1.03 × 10^−7^	3 × 10^−7^
**H**	4.49 × 10^−7^	6.92 × 10^−7^	9.04 × 10^−7^	5.57 × 10^−7^	7.32 × 10^−7^	9.77 × 10^−7^	8.88 × 10^−7^	5.71 × 10^−7^	8.33 × 10^−7^
**O**	8.99 × 10^−6^	4.06 × 10^−6^	4.66 × 10^−5^	6.71 × 10^−6^	1.29 × 10^−5^	3.82 × 10^−5^	9.35 × 10^−6^	4.26 × 10^−5^	3.15 × 10^−5^
***β_1_***	3.60 × 10^−8^	9.35 × 10^−8^	7.28 × 10^−8^	6.69 × 10^−8^	7.93 × 10^−8^	5.13 × 10^−8^	7.26 × 10^−8^	2.35 × 10^−8^	8.27 × 10^−8^
***β_2_***	9.94 × 10^−8^	1 × 10^−7^	1 × 10^−7^	1 × 10^−7^	1 × 10^−7^	1 × 10^−7^	9.9 × 10^−8^	1 × 10^−7^	1 × 10^−7^
***R_s_***	1.80	6.68	7.68	1.80	5.47	5.96	9.62	9.73	8.48
***R_CT_***	425	200	380	1000	300	280	500	530	400
***Q_dl_***	1 × 10^−7^	1.08 × 10^−4^	3.02 × 10^−4^	6.76 × 10^−4^	1.62 × 10^−4^	3.90 × 10^−4^	1 × 10^−7^	1.02 × 10^−4^	2.12 × 10^−4^
***n_dl_***	0.89	1.0	0.89	0.89	0.95	0.89	0.89	0.95	0.90
***R_film_***	46.12	---	---	492.6	---	---	102.4	---	---
***Q_film_***	5.86 × 10^−5^	---	---	2.94 × 10^−5^	---	---	8.87 × 10^−5^	---	---
***n_film_***	0.8	---	---	1	---	---	0.8	---	---

*k*_1_ (mol cm^−2^ s^−1^), *k*_2_ (cm s^−1^), *k*_3_ (cm s^−1^), *k*_4_ (cm s^−1^), *k_5_* (mol cm^−2^ s^−1^), *b*_1_ (V^−1^), *b*_2_ (V^−1^), OH (mol cm^−3^), Cl (mol cm^−3^), AlCl (mol cm^−3^), H (mol cm^−3^), O (mol cm^−3^), *β*_1_ (mol cm^−2^), *β*_1_ (mol cm^−2^), *R_s_* (Ohm cm^−2^), *R_CT_* (Ohm cm^−2^), *Q_dl_* (F cm^−2^ s^n−1^), *R_film_* (Ohm cm^−2^), *Q_film_* (F cm^−2^ s^n−1^).

## Data Availability

Not applicable.
